# Reconsideration of operative indications in pancreatic neuroendocrine neoplasms

**DOI:** 10.1186/s12957-022-02834-5

**Published:** 2022-11-18

**Authors:** Kodai Abe, Minoru Kitago, Eisuke Iwasaki, Hiroshi Yagi, Yuta Abe, Yasushi Hasegawa, Shutaro Hori, Masayuki Tanaka, Yutaka Nakano, Yuko Kitagawa

**Affiliations:** 1 Department of Surgery, Keio University School of Medicine, Shinjuku-ku, Tokyo, 160-8582 Japan; 2 Department of Internal Medicine, Keio University School of Medicine, Shinjuku-ku, Tokyo, 160-8582 Japan

**Keywords:** Pancreatic neuroendocrine neoplasm, Lymph node metastasis, Function-preserving surgery, Operative indication

## Abstract

**Background:**

The incidence of pancreatic neuroendocrine neoplasm (PNEN) has been increasing. Resection is typically indicated for PNEN, regardless of its size; however, the indications for its resection are controversial. This study aimed to evaluate the treatment results of surgical resection of PNEN at our institute.

**Methods:**

In this single-center, retrospective, case-control study, 87 patients who underwent PNEN resection and 17 patients with PNEN who did not undergo surgical resection between 1993 and 2020 were included in this study. Clinical characteristics and outcomes were reviewed and statistically compared. Survival was also estimated for the patients in each cohort.

**Results:**

Seventeen patients who underwent resection (20%) had lymph node metastasis. Tumors measuring ≥ 2.0 cm and multiple lesions were identified as independent predictors for lymph node metastasis (odds ratio [OR] 17.3, 95% confidence interval [CI] 3.0–100.0, *p* = 0.001 and OR 8.7, 95% CI 1.5–52.0, *p* = 0.018, respectively). There was a significant difference in the survival curves depending on the presence or absence of lymph node metastasis (5-year overall survival 74.7% vs. 94.3%, *p* < 0.001; 5-year recurrence-free survival: 66.3% vs. 93.6%, *p* < 0.001). All 17 PNEN cases under observation with a median 8 mm (range 5–23) tumor size for a median of 34 (range 2.4–114) months showed slight morphological change with a median tumor growth rate of 0.15 mm (range 0–3.33) per year.

**Conclusion:**

Patients with tumors measuring ≥ 2.0 cm have a high probability of lymph node metastasis or recurrence, thereby requiring resection. PNEN measuring < 1.0 cm may be acceptable for observation.

**Supplementary Information:**

The online version contains supplementary material available at 10.1186/s12957-022-02834-5.

## Background

The incidence of pancreatic neuroendocrine neoplasm (PNEN) has been increasing in the USA and Japan; it is possible that more cases are being diagnosed due to advancements in diagnostic imaging [1, 2]. PNENs have various degrees of differentiation (neuroendocrine tumor G1–3, and neuroendocrine carcinoma) and are also classified as hormone-producing (functional) or non-functional tumors, each with different grades of malignancy [3]. This has resulted in controversies regarding the indications for surgical treatment of PNEN. According to the latest Japanese Guidelines for Neuroendocrine Tumors of the Pancreas and Gastrointestinal Tract, all PNENs are indicated for surgical resection [4]. However, the latest National Comprehensive Cancer Network guidelines and the European guidelines proposed that function-preserving surgery (enucleation or partial pancreatectomy) and strict observation may be acceptable for tumors < 2 cm in size [5, 6].

Some environmental risk factors for PNEN have been identified; for example, type 2 diabetes for females and cardiovascular diseases for males [7, 8]. Furthermore, metabolic syndrome or non-alcoholic fatty liver disease have been reported to be contributed to worsening PNEN pathogenicity [9], and various factors that determine the prognosis of PNEN have been reported; these include tumor size and the presence or absence of lymph node metastases, all of which are closely related to surgical treatment strategies [10, 11]. However, there is no consensus regarding whether lymph node dissection should be performed depending on the size of the tumor or indications for function-preserving surgery and observational follow-up [12].

Herein, we analyzed and evaluated the treatment results for surgical resection of PNEN at our institute and proposed treatment strategies based on our analysis.

## Methods

### Study design

This was a single-center, retrospective, case-control study and was conducted according to the principles of the Declaration of Helsinki after approval from our Institutional Review Board (authorization number: 20120443).

### Patients and data collection

This study included 87 patients for whom PNEN resection was performed at our institute between January 1993 and December 2020. Furthermore, we also enrolled 17 patients undergoing follow-up for PNEN who did not undergo resection at our institute during the same period. Seven (8.0%) resected patients with PNEN who already had distant unresectable metastases were also included. All of them had liver metastasis. No patients with PNEN were observed to have distant metastasis. Patients’ clinical details were collected from our medical records. The short- and long-term outcomes including surgical techniques were retrospectively reviewed. For patients with more than one PNEN, the largest diameter of the tumors was adopted as the tumor size.

### Treatment protocol for PNEN

**Fig. 1:**
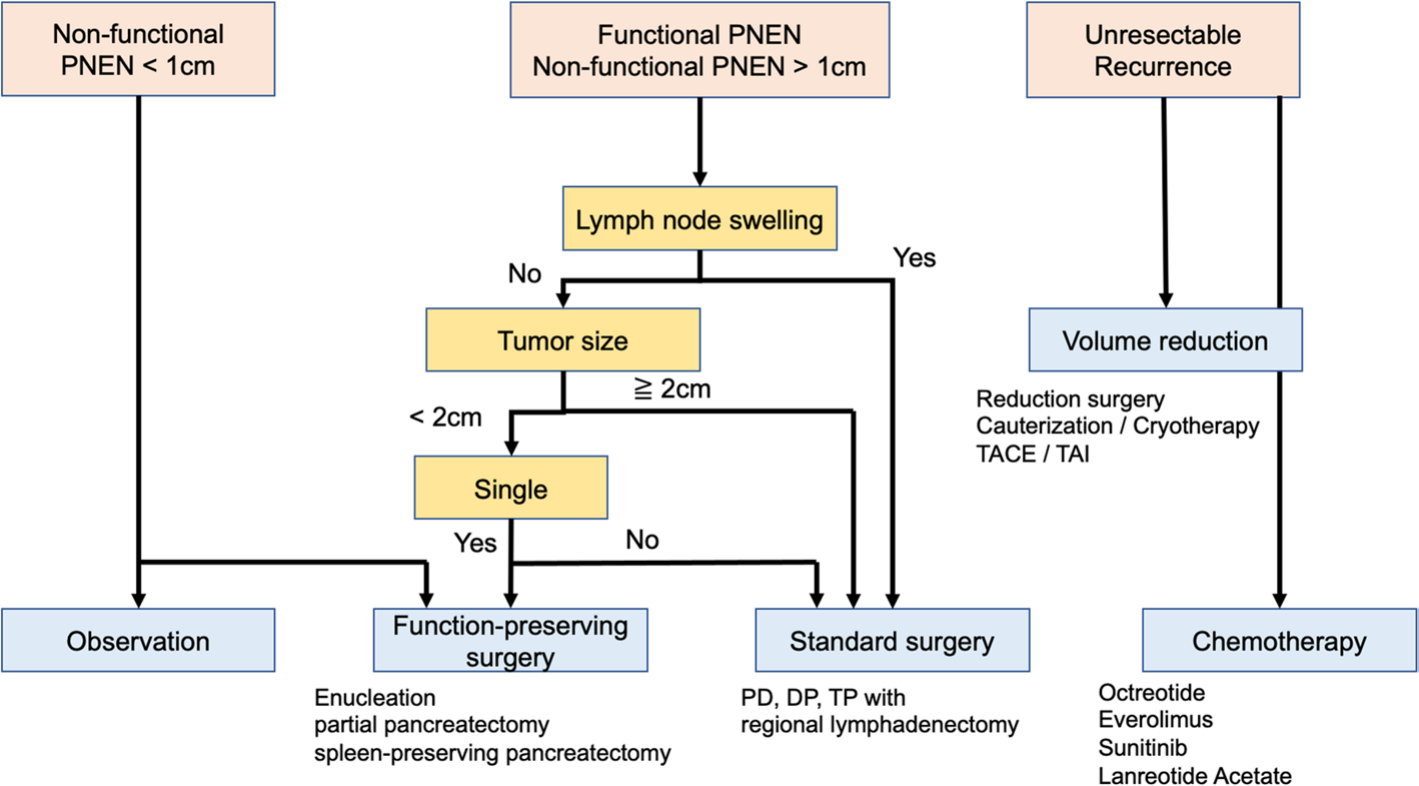
Treatment flowchart of pancreatic neuroendocrine neoplasms at our institute. We have set up this protocol with reference to the latest Japanese Guidelines for Neuroendocrine Tumors of the Pancreas and Gastrointestinal Tract and are using it in clinical practice. PNEN, pancreatic neuroendocrine neoplasm; TACE, transarterial chemoembolization; TAI, transcatheter arterial infusion chemotherapy; PD, pancreaticoduodenectomy; DP, distal pancreatectomy; TP, total pancreatectomy

Our treatment protocol for PNEN is summarized in Fig. 1. This protocol is based on the latest Japanese Guidelines for Neuroendocrine Tumors of the Pancreas and Gastrointestinal Tract [3]. Standard surgery (pancreaticoduodenectomy, distal pancreatectomy, or total pancreatectomy) was performed if the tumor was visualized to be ≥ 2.0 cm in size and enlarged lymph nodes were noted. Even if there were no enlarged lymph nodes but the tumor size was ≥ 2.0 cm, standard surgery was performed. If the tumor measured 1.0–2.0 cm in size and solitary, function-preserving surgeries such as enucleation, partial resection, and central pancreatectomy were considered. If a tumor was sized < 1.0 cm in size, function-preserving surgery was selected sometimes, but careful observation was also considered after consultation with internal medicine specialists. In patients with advanced PNEN with distant metastasis, radical surgery was performed if the patient’s general condition and surgical technique were appropriate; additionally, chemotherapy was administered. We offered treatment to patients diagnosed with PNEN according to the protocol in Fig. 1.

### Statistical analyses

The statistical review of the study was performed by a biomedical statistician. Categorical variables between the two groups were compared using chi-squared and Fisher’s exact tests. The Mann–Whitney *U* test was performed to compare quantitative variables to determine the distribution of the data. An exact logistic regression analysis was used to examine the odds ratio for lymph node metastasis. Tumor size (≥ 2.0 cm or < 2.0 cm), and the number of lesions (multiple or single) were considered for inclusion in a multivariate model. Statistical analyses were performed using SPSS Statistics for Mac (version 25.0; IBM, Armonk, NY, USA). Statistical significance was set at two-sided *p* values of < 0.05. The Kaplan–Meier analysis and log-rank test were used to estimate the survival of the cohort patients. Proportional hazard analysis was performed using a Cox regression model.

## Results

### Patient’s characteristics for resected PNEN

The clinical characteristics of patients with resected PNEN are shown in Additional file 1. The median tumor size was 1.7 cm (range 0.3–13.5 cm). There were eight and five patients with multiple endocrine neoplasm type 1 and von Hippel–Lindau disease, respectively. Four out of 8 MEN1 PNEN patients and one out of 5 VHL PNEN patients had multiple lesions, and 2 MEN1 patients and one VHL patient experienced postoperative recurrence (Additional file 2). Functional PNEN was pathologically determined in 26 patients (29.9%). All patients with insulinoma had hypoglycemia as an initial symptom. We experienced 2 patients with pathologically diagnosed glucagonoma; one had epigastric pain but the other had no symptoms. The number of patients with PNEN resection increased over time (Additional file 3). Furthermore, the number of tumors measuring < 2 cm (especially < 1 cm) increased. Eleven patients (12.6%) had multiple lesions. Middle pancreatectomy, enucleation, and partial resection were performed as function-preserving surgeries in 11 patients who were selectively treated for small tumors measuring < 2.0 cm. The long-term outcomes of these patients were favorable. However, grade B or higher pancreatic fistulae were observed in five patients (44.4%). Sixteen patients (18.4%) experienced recurrence after surgery, mainly as liver metastasis. Before 2000, we performed hepatic arterial injection chemotherapy for liver metastasis; however, since 2000, we have used somatostatin analogs (SSA) or hepatic resection for liver metastasis.

### Risk factor analysis for lymph node metastasis, recurrence, and death of resected PNEN

Tumor size was significantly larger in patients with lymph node metastasis than in those without (median 4.0 cm [range 0.8–13.5 cm] vs. 1.5 cm [range 0.3–3.7], *p* < 0.001) (Table 1). Furthermore, the occurrence of multiple lesions was significantly higher in patients with lymph node metastasis than in those without (29.4% vs. 8.6%, *p* = 0.020). When a receiver operating characteristic curve for tumor size was drawn with and without lymph node metastasis, a cut-off value of 2.0 cm (area under the curve 0.835) was calculated (Additional file 4). Multivariate logistic regression analysis also revealed that tumor measuring ≥ 2.0 cm and the presence of multiple lesions were independent risk factors for lymph node metastasis (tumor size ≥ 2.0 cm: odds ratio [OR] 10.8, 95% confidence interval [CI] 3.0–100.0, *p* = 0.001; multiple lesions: OR 8.7, 95% CI 1.5–52.0, *p* = 0.018) (Table 2). However, metastatic lymph nodes were confined to the periphery of the tumor body in all resected PNEN patients with positive lymph node metastasis.

**Table 1:** Risk factors for lymph node metastasis of pancreatic neuroendocrine neoplasm

	Univariate analysis				Multivariate analysis		
	Lymph node positive (*n* = 17)	Lymph node negative (*n* = 70)	*P* value		Odds ratio	95% CI	*P* value
Age (median, years)	50 (34–78)	62 (18–84)	0.113				
Male / Female	4/13	38/32	0.110				
Tumor size (median, cm)	4.0 (0.8–13.5)	1.5 (0.3–3.7)	< 0.001	≧ 2.0 cm	10.8	3.0–100.0	0.001
Multiple lesions	5 (29.4%)	6 (8.6%)	0.020		8.7	1.5–52.0	0.018
Hereditary syndrome	3 (17.6%)	10 (14.3%)	0.727				
Preoperative symptoms	7 (41.2%)	18 (25.7%)	0.206				
Pathologically functional PNEN	5 (29.4%)	20 (28.6%)	0.945				
Grade (G1 vs G2/G3)	6 (35.3%)	31 (44.3%)	0.444				
Ly (0/1 vs 2/3)	2 (11.8%)	5 (7.1%)	0.540				
V (0/1 vs 2/3)	3 (17.6%)	13 (18.6%)	0.910				
Ne (0/1 vs 2/3)	2 (11.8%)	5 (7.1%)	0.540				
Recurrence	9 (52.9%)	7 (10.0%)	< 0.001				

*PNEN* pancreatic neuroendocrine neoplasm, *Ly* lymphatic invasion, *V* vascular invasion, *Ne* neural invasion

**Table 2:** Preoperative and pathological risk factors for survival and recurrence of PNEN

	Hazard ratio	95% CI	*P* value
Preoperative risk factors			
Risk of recurrence			
Age (years)	1.0	0.98–1.06	0.315
Male/female	1.5	0.45–4.95	0.510
Tumor size ≥ 2.0 cm	22.7	2.72–188.90	0.004
Multiple lesions	5.1	0.83–30.85	0.078
Hereditary syndrome	0.7	0.15–3.38	0.665
Preoperative symptoms	1.2	0.33–4.24	0.788
Risk of death			
Age (years)	1.0	0.99–1.10	0.096
Male/female	0.8	0.21–2.82	0.687
Tumor size ≥ 2.0 cm	20.0	2.18–183.38	0.008
Multiple lesions	5.6	0.84–37.28	0.076
Hereditary syndrome	0.9	0.13–6.05	0.898
Preoperative symptoms	0.7	0.13–3.45	0.632
Pathological risk factors			
Risk of recurrence			
Pathologically functional PNEN	0.1	0.00–1.54	0.094
Grade (G1 vs G2/G3)	1.3	0.32–4.98	0.732
Ly (0/1 vs 2/3)	0.3	0.01–7.97	0.492
V (0/1 vs 2/3)	3.0	0.50–18.68	0.230
Ne (0/1 vs 2/3)	5.2	0.72–37.49	0.102
Lymph node metastasis	9.5	2.85–31.43	< 0.001
Risk of death			
Pathologically functional PNEN	0.5	0.09–2.91	0.452
Grade (G1 vs G2/G3)	0.6	0.11–3.15	0.535
Ly (0/1 vs 2/3)	2.2	0.04–119.40	0.698
V (0/1 vs 2/3)	0.7	0.04–14.21	0.825
Ne (0/1 vs 2/3)	1.0	0.03–38.52	0.986
Lymph node metastasis	8.0	1.95–32.59	0.004

*PNEN* pancreatic neuroendocrine neoplasm, *Ly* [lymphatic invasion], *V* [vascular invasion], *Ne* [neural invasion]

We further analyzed the hazard ratio of recurrence and death for resected PNEN using preoperative and postoperative parameters (Table 2). In the preoperative parameters, the risk of recurrence and death was significantly higher for tumors measuring ≥ 2.0 cm (hazard ratio [HR] for recurrence 22.7, 95% CI 2.72–188.90, *p* = 0.004; HR for death 20.0, 95% CI 2.18–183.38, *p* = 0.008). In the postoperative pathological parameters, lymph node metastasis was found to have a significantly higher risk of recurrence and death than the other parameters (HR for recurrence 9.5, 95% CI 2.85–31.43, *p* < 0.001; HR for death 8.0, 95% CI 1.95–32.59, *p* = 0.004).

### Survival analysis of resected PNEN

**Fig. 2:**
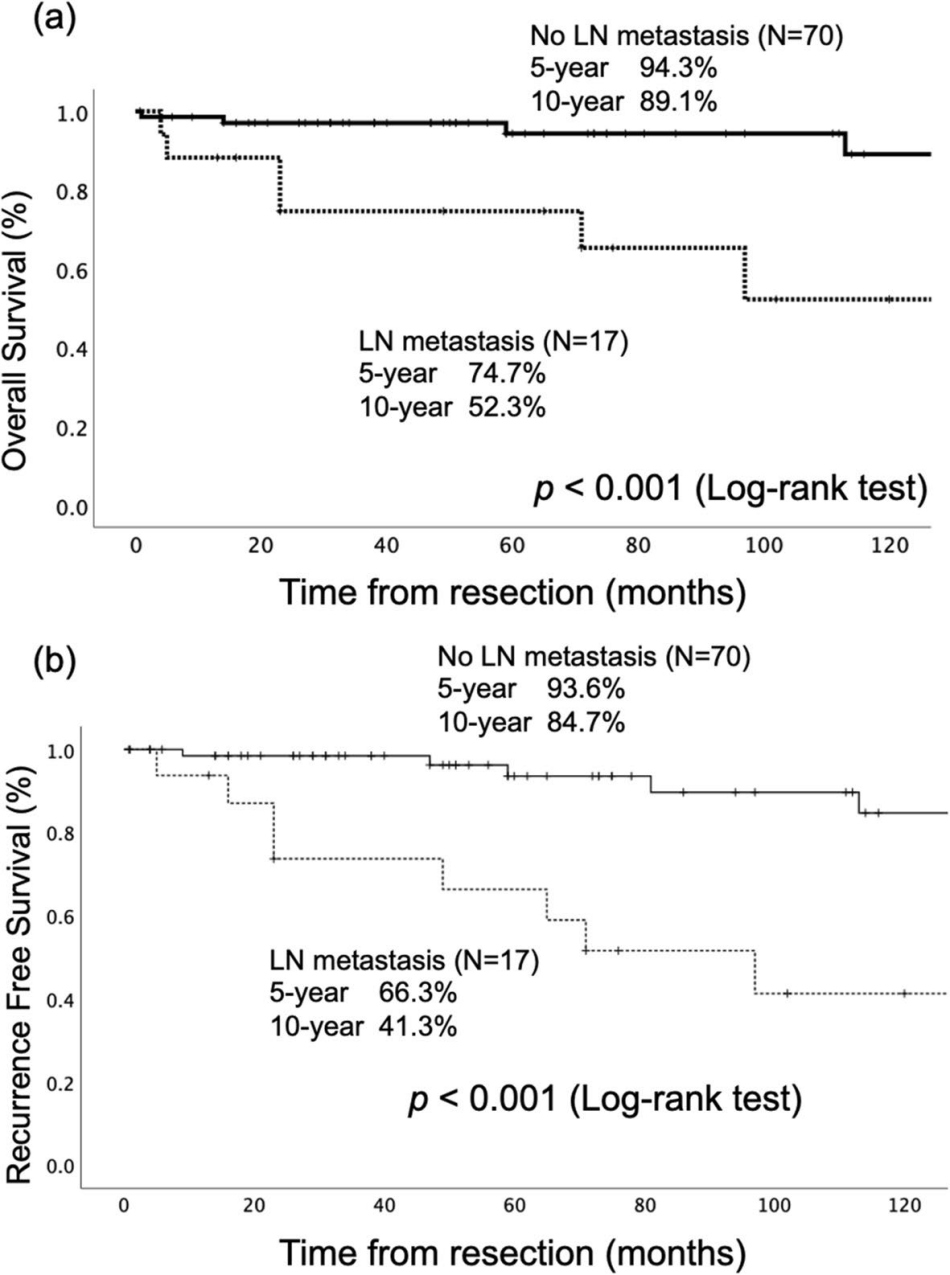
Survival analysis for patients with resected pancreatic neuroendocrine neoplasms with or without lymph node metastasis. **a** Overall survival showed PNEN patients without lymph node metastasis had a more favorable prognosis than those with positive lymph node metastasis. **b** Recurrence-free survival also showed PNEN patients without lymph node metastasis had more favorable outcomes than those with positive lymph node metastasis. PNEN, pancreatic neuroendocrine neoplasm; LN, lymph node

**Fig. 3:**
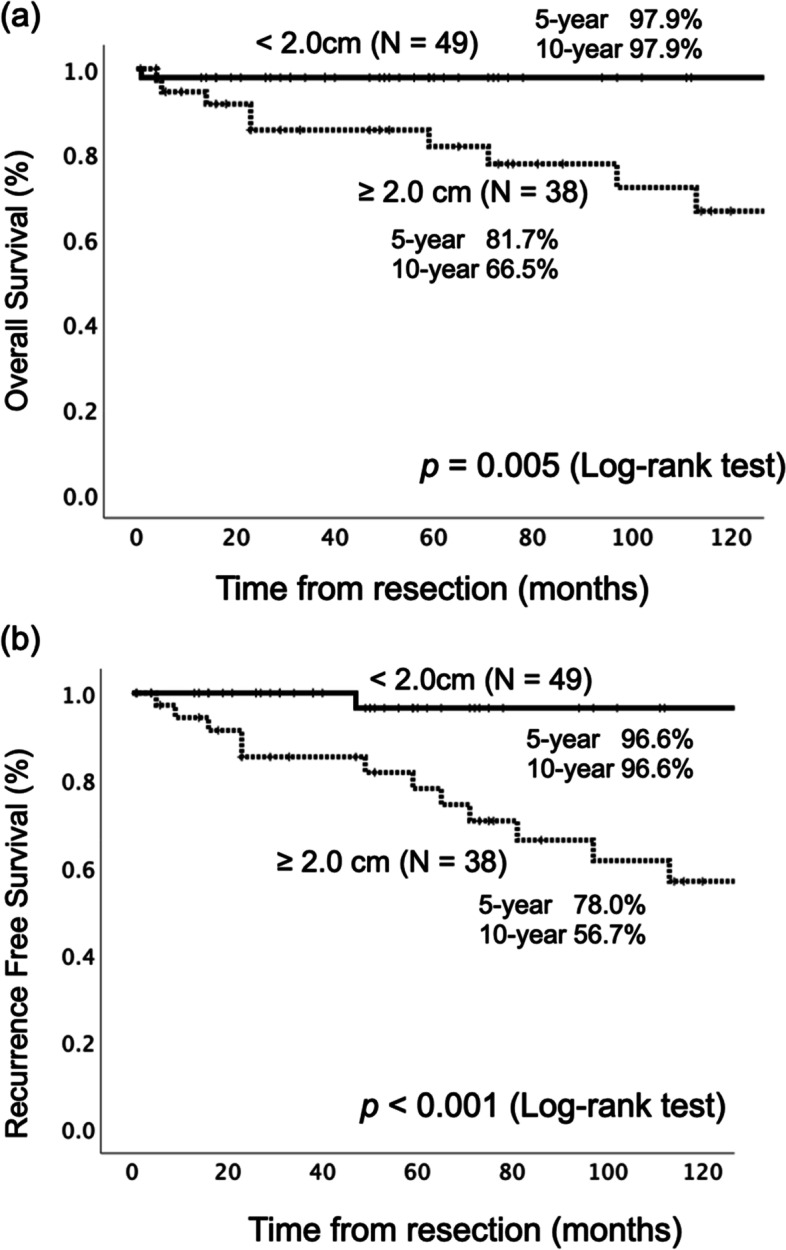
Survival analysis for patients with resected pancreatic neuroendocrine neoplasms depending on tumor size. **a** Overall survival showed PNEN patients with metastasized tumor measuring < 2.0 cm had a more favorable prognosis than those with tumor size ≥ 2.0 cm. **b** Recurrence-free survival also showed PNEN patients with tumor measuring < 2.0 cm in size had more favorable outcomes than those with tumor measuring ≥ 2.0 cm. PNEN, pancreatic neuroendocrine neoplasm

The 5- and 10-year overall survival rates were 90.5% and 80.7%, respectively (Additional file 5). Recurrence was observed in 16 patients, which included 11 liver metastases, 3 lymph node metastases, 1 bone metastasis, and 1 remnant pancreas recurrence. However, Kaplan–Meier analysis showed that there was a significant difference in the overall survival and recurrence curves depending on the presence or absence of lymph node metastasis (Fig. 2A, B; log-rank test, *p* < 0.001). There was also a significant difference in the overall survival and recurrence curves depending on the tumor size ≥ 2.0 or < 2.0 cm (Fig. 3A, B; log-rank test, *p* = 0.005 and *p* < 0.001, respectively).

### Patients under observation for PNEN

There were 17 additional patients with PNEN who were undergoing follow-up at our institute (Table 3). Case 4 in Table 3 was diagnosed as MEN1 at diagnosis of PNEN. These patients were diagnosed with PNEN by radiologists and endoscopists using enhanced computed tomography or endoscopic ultrasound. As shown in Additional file 6, the median tumor size was 8 mm (range 5–23 mm) and median tumor growth rate in these patients was 0.15 mm (range 0–3.33 mm) per year, suggesting that the tumor size remained almost the same despite the long-term follow-up. All of them were still alive with no disease progression at the last follow-up visit. In particular, the four patients diagnosed with G1 (2 patients) or G2 (2 patients) tumors using endoscopic ultrasound fine-needle aspiration did not show any significant change in tumor size over time (Table 3). None of them receive chemotherapy or other treatments for PNEN.

**Table 3:** Observed PNEN patients at our institute

Case	Age	Sex	Size (mm)	Morphological change	Growth rate (mm/year)	EUS-FNA	Location	NSE	Overall survival (months)	Outcome
1	53	Female	11	8 mm→11 mm	0.31	–	Tail	10.1	114.3	Alive
2	78	Male	8	8 mm→8 mm	0	–	Body	14.5	55.8	Alive
3	70	Male	9	8 mm→9 mm	0.40	G2	Head	9.3	30.3	Alive
4	51	Male	① 17 (tail) ②12 (body) ③14 (head) ④ 8 (head)	①17 mm→16 mm ②12 mm→13 mm ③14 mm→16 mm ④8 mm→8.7 mm	①-0.41 ②0.41 ③0.82 ④0.29	G1	Multiple	10.8	29.2	Alive
5	60	Male	12	12 mm→12 mm	0	–	Head	14.4	103.0	Alive
6	66	Male	9	9 mm→9 mm	0	–	Head	9.4	46.5	Alive
7	35	Male	8	8 mm→10 mm	0.84	–	Head	N.E.	28.5	Alive
8	86	Female	15	15 mm→17 mm	0.83	–	Tail	N.E.	29.0	Alive
9	35	Female	8	8 mm→8 mm	0	–	Body	N.E.	56.3	Alive
10	70	Male	7	7 mm→7 mm	0	–	Body	N.E.	58.1	Alive
11	73	Male	5	5 mm→8 mm	0.99	–	Head	N.E.	36.5	Alive
12	48	Male	7	7 mm→7 mm	0	–	Body	N.E.	13.3	Alive
13	60	Female	7	7 mm→7 mm	0	–	Body	N.E.	38.9	Alive
14	78	Male	7	7 mm→8 mm	0.35	–	Body	N.E.	34.7	Alive
15	71	Female	13	13 mm→13 mm	0	G2	Tail	N.E.	16.0	Alive
16	88	Male	23	23 mm→25 mm	3.33	–	Body	N.E.	7.2	Alive
17	62	Female	19	19 mm→19 mm	0	G1	Head	N.E.	2.4	Alive

*PNEN* pancreatic neuroendocrine neoplasm, *EUS* endoscopic ultrasound, *FNA* fine-needle aspiration, *NSE* neuro-specific enolase, *N.E*. not examined

## Discussion

The current guidelines and results of previous studies regarding indications for surgical treatment of PNEN provide inconsistent recommendations. Some studies suggest that lymph node metastasis in PNEN does not affect prognosis [13, 14], while other studies suggest that lymph node metastasis is a defining factor [15]. Our results showed that patients with tumors measuring ≥ 2.0 cm had a higher frequency of lymph node metastasis than those with tumors measuring < 2.0 cm. Although lymph node metastasis was only observed around the main tumor body, there was a significant difference in survival prognosis between these patients. Therefore, patients with PNEN who have a large tumor size (≥ 2.0 cm) should undergo lymph node dissection with standard surgery.

Evidence shows that although long-term outcomes of function-preserving surgeries are favorable, delayed recurrence and perioperative complications are common after such surgeries [16, 17]. Hence, we believe that adequate perioperative management and follow-up are necessary for patients undergoing function-preserving surgeries.

Recent studies have suggested that observational follow-ups may be acceptable for small PNENs owing to their low malignancy risk [18–20]. Pancreatectomy for PNEN is one of the risk factors for postoperative pancreatic fistulae because of the soft pancreas and narrow main pancreatic duct. Our results also indicated that 12 (13.8%) patients had postoperative pancreatic fistulae (5 received function-preserving surgery and 7 received standard pancreatectomy with lymphadenectomy). It is preferable to avoid pancreatectomy if observation is appropriate. However, lymph node metastasis has been observed in patients with resected glucagonoma (tumor size 0.7 cm) and gastrinoma (tumor size 0.8 and 1.8 cm), suggesting that follow-up alone is not acceptable for functional PNEN. This result supports the importance of lymphadenectomy for gastrinoma or glucagonoma [21, 22]. Although possible, it is not easy to identify gastrinomas and glucagonomas with poor prognosis from clinical symptoms alone [23, 24]. Hayashi et al. [25] and our group [26] showed that selective arterial calcium injection (SACI) is useful for diagnosing small functional PNENs that cannot be captured on images. Therefore, if glucagonoma or gastrinoma is diagnosed or strongly suspected by SACI, standard surgery with lymph node dissection should be performed even if the tumor size is < 1.0 cm.

Finally, we often experienced recurrences of PNEN after surgery, mainly as liver metastasis. In particular, PNENs with genetic syndromes such as MEN1 and VHL, are likely to be multiple with higher risk of recurrence than solitary PNENs. As the usefulness of SSA is increasingly demonstrated especially for PNEN with MEN1 [27], techniques to combine SSA with other treatment modalities including surgical resection and genetic testing or MEN1/VHL screening before treatment, will become increasingly important in the future (Additional file 7).

### Limitations of the study

Our study has the following limitations. It was a retrospective study conducted at a single institute with a small sample size, but the statistical analysis provided important findings. Although we considered not only resected cases but also observed cases, it is necessary to launch a multicenter prospective randomized controlled trial to unify these cases and determine the indication for surgery. In any case, long-term follow-up of the observed PNEN is essential. Tissue examination was not performed in all observed patients with PNEN. Furthermore, we were unable to clarify why a deviation between lymph node metastasis and grade classification emerged. According to Table 1, 44% of patients with grade 2 or 3 PNEN had no lymph node metastasis. Finally, nuclear fission and Ki-67 level have been considered the important criteria for the prognosis of PNEN, but our findings did not corroborate this; therefore, this should be clarified in future research.

## Conclusions

Patients with tumors measuring ≥ 2.0 cm are likely to have lymph node metastasis or recurrence, suggesting the need for standard surgery. PNEN measuring < 1.0 cm may be acceptable for careful observation. Gastrinoma and glucagonoma frequently metastasize to the lymph nodes, even when the tumor size is small, suggesting the need for resection with lymphadenectomy.

## Supplementary Information

**Additional file 1.** Clinical characteristics of patients with pancreatic neuroendocrine neoplasms.

**Additional file 2.** PNEN patients with genetic syndromes.

**Additional file 3.** Time transition of resected PNEN patients during study period. PNEN, pancreatic neuroendocrine neoplasm.

**Additional file 4.** Receiver operating characteristic curve of tumor size with and without lymph node metastasis in pancreatic neuroendocrine neoplasm.

**Additional file 5. **Overall survival analysis for patients with resected pancreatic neuroendocrine neoplasm. We analyzed resected PNEN survival using Kaplan-Meyer method, showing that both overall and recurrence free survival were favorable. OS, overall survival; RFS, recurrence free survival.

**Additional file 6.** Review of observed PNEN cases.

**Additional file 7.** Updated treatment flowchart of PNEN according to our findings.

## Data Availability

All data generated or analyzed during this study are included in this published article [and its supplementary information files].

## Abbreviations

PNEN: Pancreatic neuroendocrine neoplasm
OR: Odds ratio
CI: Confidence interval
HR: Hazard ratio
SSA: Somatostatin analog
SACI: Selective arterial calcium injection
