# Supplementary material for: Reconsideration of operative indications in pancreatic neuroendocrine neoplasms
Source: World J Surg Oncol. 2022 Nov 18;20:366. doi: 10.1186/s12957-022-02834-5 (PMC9673351; doi:10.1186/s12957-022-02834-5)
Supplement: Supplementary file 1 — Additional file 1. Clinical characteristics of patients with pancreatic neuroendocrine neoplasms. [file 12957_2022_2834_MOESM1_ESM.docx]

**Additional File 1. Clinical characteristics of patients with pancreatic neuroendocrine neoplasms**

| N=87 |  |
| --- | --- |
| Age (median, years) | 58 (18-84) |
| Sex (male / female) | 51 / 36 |
| Tumor size (median, cm) | 1.7 (0.3-13.5) |
| MEN-1/ VHL | 8 / 5 |
| Multiple lesions | 11 (12.6%) |
| Symptoms | 29 (33.3%) |
|  | (pain in the right quadrant, abdominal tumor, liver dysfunction, jaundice, black stools, hypoglycemia, etc.) |
| Pathological functional PNEN | 26 (29.9%) |
|  | (insulinoma, 17; gastrinoma, 4; glucagonoma, 2; serotonin, 2; somatostatinoma, 1) |
| Localization | head, 30; body, 19; tail, 27; multiple, 11 |
| Surgical procedure | pancreaticoduodenectomy, 28; distal pancreatectomy, 45; total pancreatectomy 3; enucleation, 7; partial resection, 2; middle pancreatectomy, 2 |
| MEN-1, multiple endocrine neoplasm type 1; VHL, von Hippel-Lindau disease; PNEN, pancreatic neuroendocrine neoplasm. | |
