# Supplementary material for: Reconsideration of operative indications in pancreatic neuroendocrine neoplasms
Source: World J Surg Oncol. 2022 Nov 18;20:366. doi: 10.1186/s12957-022-02834-5 (PMC9673351; doi:10.1186/s12957-022-02834-5)
Supplement: Supplementary file 2 — Additional file 2. PNEN patients with genetic syndromes. [file 12957_2022_2834_MOESM2_ESM.docx]

**Additional File 2. PNEN patients with genetic syndromes**

| Case | Age | Sex | Genetic syndrome | Multiple lesions | Distant metastasis | Functional lesion | Operative procedure | Recurrence | Treatment for recurrence | Overall survival  (months) | Outcome |
| --- | --- | --- | --- | --- | --- | --- | --- | --- | --- | --- | --- |
| 1 | 39 | male | MEN1 | No | No | No | DP | No | - | 245.0 | Alive |
| 2 | 40 | male | MEN1 | No | No | No | DP | No | - | 29.0 | Alive |
| 3 | 40 | female | VHL | No | No | No | PP | No | - | 224.0 | Alive |
| 4 | 45 | male | VHL | No | No | No | TP | Yes (Liver) | N.D. | 113.0 | Dead |
| 5 | 56 | female | MEN1 | Yes | No | Gastrinoma | TP | Yes (Liver) | TACE / Octreotide | 97.0 | Dead |
| 6 | 32 | female | VHL | Yes | No | Somatostatinoma | PD | No | - | 172.0 | Alive |
| 7 | 70 | male | MEN1 | Yes | No | Insulinoma | EN | No | - | 112.0 | Alive |
| 8 | 38 | male | MEN1 | Yes | No | Insulinoma | DP | Yes (Lymph node) | Octreotide | 59.0 | Dead |
| 9 | 57 | male | VHL | No | No | No | TP | No | - | 114.0 | Alive |
| 10 | 43 | male | MEN1 | Yes | No | Gastrinoma | PP | No | - | 102.0 | Alive |
| 11 | 18 | male | MEN1 | No | No | Insulinoma | DP | No | - | 86.0 | Alive |
| 12 | 51 | female | VHL | No | No | No | PD | No | - | 33.0 | Alive |
| 13 | 34 | male | MEN1 | Yes | Liver | No | PD | No | - | 16.0 | Alive |
| PNEN, pancreatic neuroendocrine neoplasm; MEN1, multiple endocrine neoplasm type1; VHL, von-Hippel Lindau disease; DP, distal pancreatectomy; PP, partial pancreatectomy; TP, total pancreatectomy; PD, pancreaticoduodenectomy; EN, enucleation; N.D., not described; TACE, transcatheter arterial chemoembolization. | | | | | | | | | | | |
