# Supplementary material for: Reconsideration of operative indications in pancreatic neuroendocrine neoplasms
Source: World J Surg Oncol. 2022 Nov 18;20:366. doi: 10.1186/s12957-022-02834-5 (PMC9673351; doi:10.1186/s12957-022-02834-5)
Supplement: Supplementary file 3 — Additional file 3. Time transition of resected PNEN patients during study period. PNEN, pancreatic neuroendocrine neoplasm. [file 12957_2022_2834_MOESM3_ESM.docx]

**Additional File 3. Time transition of resected PNEN patients during study period. PNEN, pancreatic neuroendocrine neoplasm.**

**(a) Total number of PNEN patients**

**(b) Depending on the tumor size (< 1 cm, 1-2 cm, > 2 cm)**
