# Supplementary material for: Reconsideration of operative indications in pancreatic neuroendocrine neoplasms
Source: World J Surg Oncol. 2022 Nov 18;20:366. doi: 10.1186/s12957-022-02834-5 (PMC9673351; doi:10.1186/s12957-022-02834-5)
Supplement: Supplementary file 4 — Additional file 4. Receiver operating characteristic curve of tumor size with and without lymph node metastasis in pancreatic neuroendocrine neoplasm. [file 12957_2022_2834_MOESM4_ESM.docx]

**Additional File 4. Receiver operating characteristic curve of tumor size with and without lymph node metastasis in pancreatic neuroendocrine neoplasm.**

AUC, area under the curve.

**
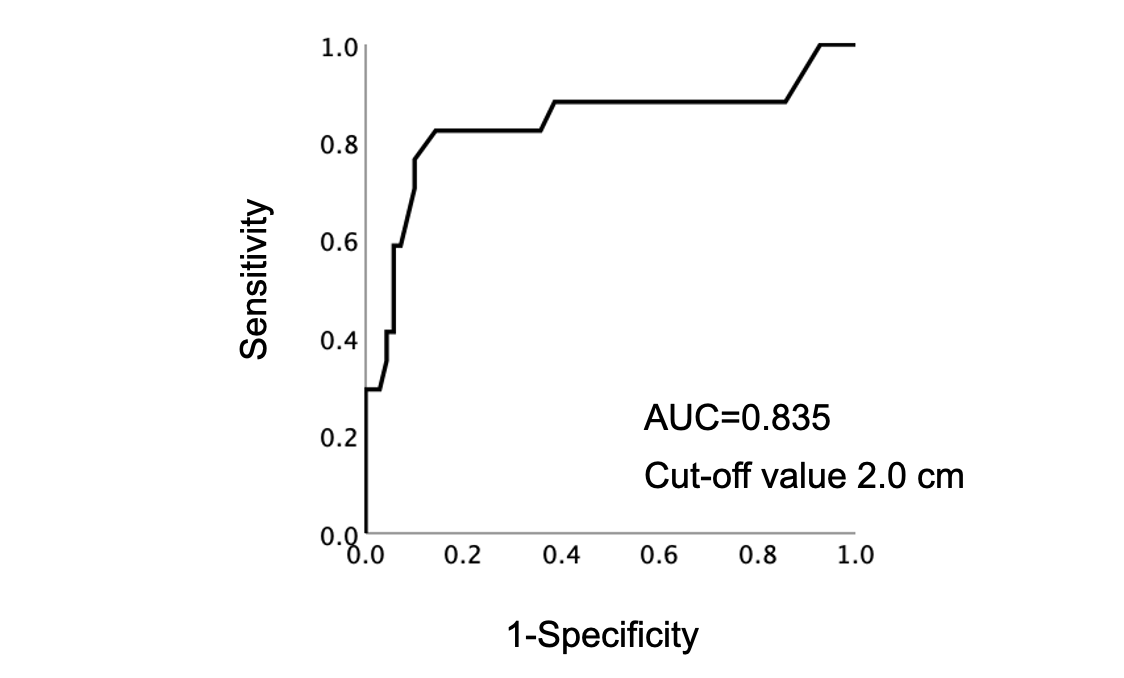
**
