# Supplementary material for: Reconsideration of operative indications in pancreatic neuroendocrine neoplasms
Source: World J Surg Oncol. 2022 Nov 18;20:366. doi: 10.1186/s12957-022-02834-5 (PMC9673351; doi:10.1186/s12957-022-02834-5)

**Additional File 5. Overall survival analysis for patients with resected pancreatic neuroendocrine neoplasm. We analyzed resected PNEN survival using Kaplan-Meyer method, showing that both overall and recurrence free survival were favorable. OS, overall survival; RFS, recurrence free survival.**


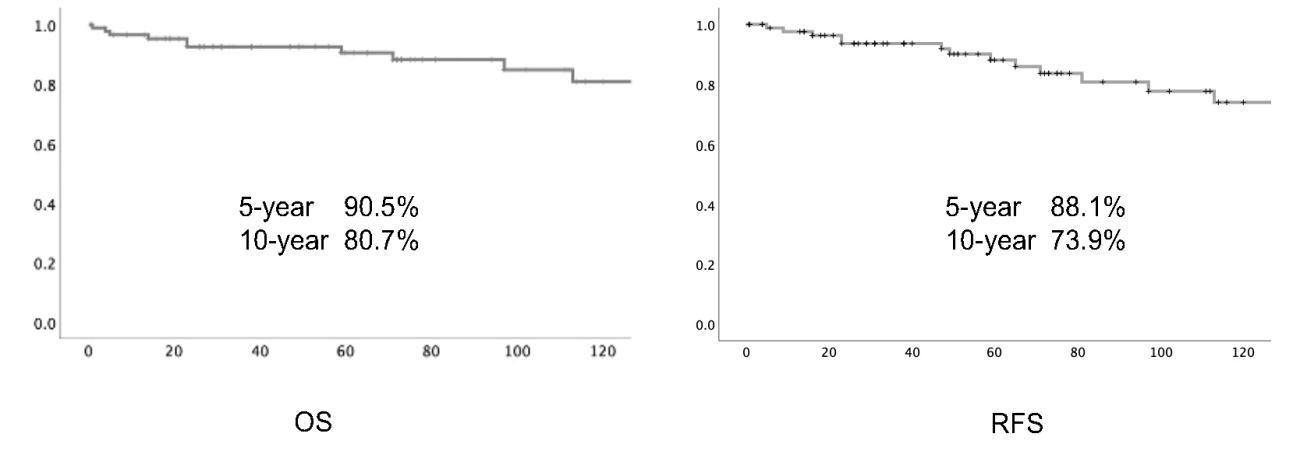

Supplement: Supplementary file 5 — Additional file 5. Overall survival analysis for patients with resected pancreatic neuroendocrine neoplasm. We analyzed resected PNEN survival using Kaplan-Meyer method, showing that both overall and recurrence free survival were favorable. OS, overall survival; RFS, recurrence free survival. [file 12957_2022_2834_MOESM5_ESM.docx]
