# Supplementary material for: Reconsideration of operative indications in pancreatic neuroendocrine neoplasms
Source: World J Surg Oncol. 2022 Nov 18;20:366. doi: 10.1186/s12957-022-02834-5 (PMC9673351; doi:10.1186/s12957-022-02834-5)
Supplement: Supplementary file 6 — Additional file 6. Review of observed PNEN cases. [file 12957_2022_2834_MOESM6_ESM.docx]

**Additional File 6. Review of observed PNEN cases**

|  | N=17 |
| --- | --- |
| Age (median, years) | 66 (35-88) |
| Sex (male / female) | 11 / 6 |
| Tumor size (median, mm) | [Initial] 8 (5-23)→[Current] 9.5 (7-25) |
| Hypervascular lesion | 15 (88.2%) |
| EUS-FNA | G1: 2, G2: 2 |
| Observation periods (median, months) | 34 (2.4-114) |
| Death | 0 |
| PNEN, pancreatic neuroendocrine neoplasm; EUS, endoscopic ultrasound; FNA, fine needle aspiration. | |
