# Supplementary figures and images for: Reconsideration of operative indications in pancreatic neuroendocrine neoplasms
Source: World J Surg Oncol. 2022 Nov 18;20:366. doi: 10.1186/s12957-022-02834-5 (PMC9673351; doi:10.1186/s12957-022-02834-5)

**Additional File 7. Updated treatment flowchart of PNEN according to our findings**


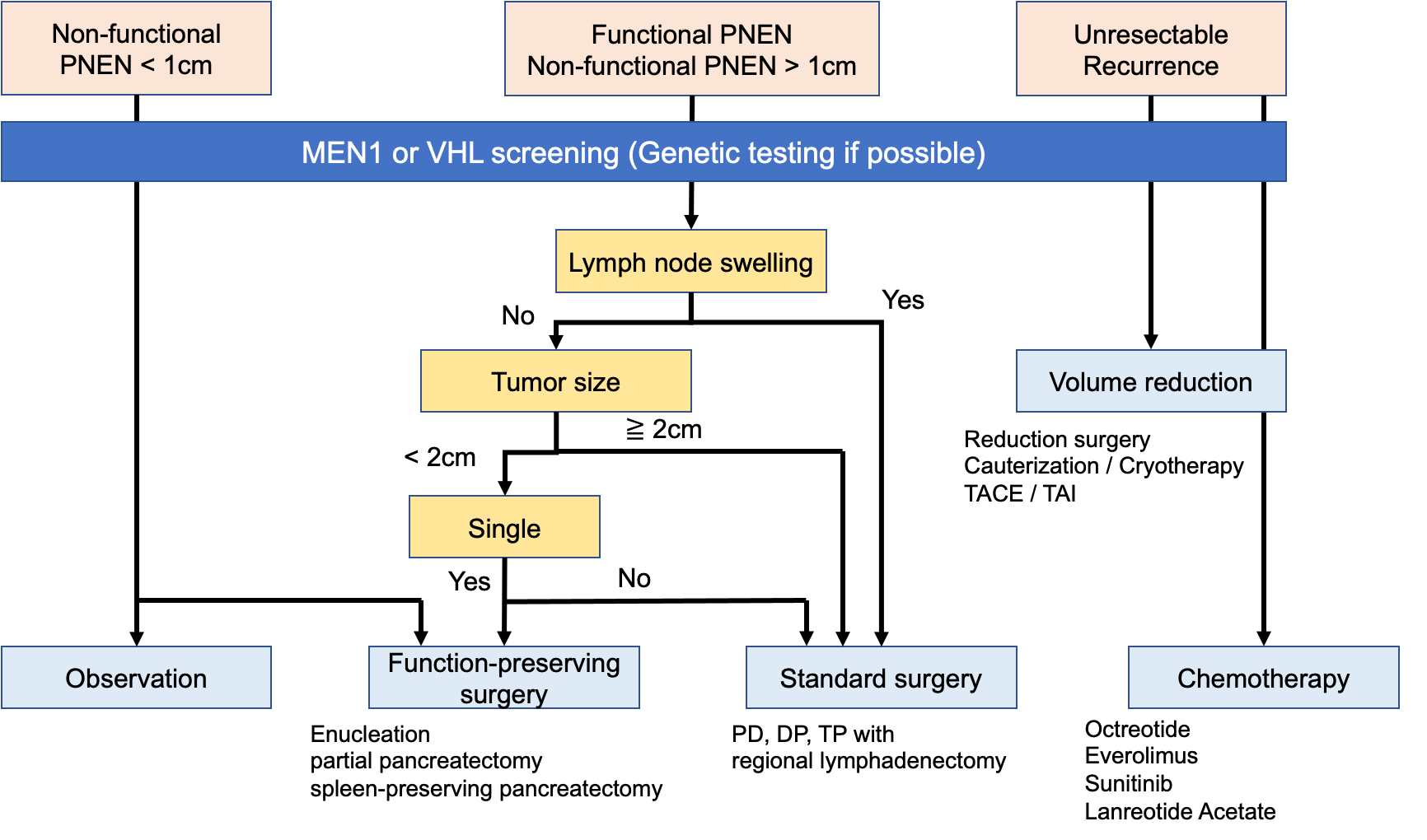

Supplement: Supplementary file 7 — Additional file 7. Updated treatment flowchart of PNEN according to our findings. [file 12957_2022_2834_MOESM7_ESM.docx]
